# Differential Effects of Pre-Fermentation with Different Lactic Acid Bacteria Strains on the Structure, Functionality, and Flavor of Semi-Dry Milled Glutinous Rice Flour

**DOI:** 10.3390/foods15142496

**Published:** 2026-07-14

**Authors:** Jingyi Zhang, Shan Shan, Shan Zhang, Di Yuan, Qi Wu, Bin Hong, Chuanying Ren

**Affiliations:** 1 Food Processing Research Institute, Heilongjiang Academy of Agricultural Sciences, Harbin 150086, China; 18846080235@139.com (J.Z.); 18845896856@163.com (S.S.); zhangshanfood@163.com (S.Z.); yuandi199707@163.com (D.Y.); wuqi0322@163.com (Q.W.); gru.hb@163.com (B.H.); 2 Heilongjiang Province Key Laboratory of Food Processing, Harbin 150086, China; 3 Heilongjiang Province Engineering Research Center of Whole Grain Nutritious Food, Harbin 150086, China

**Keywords:** glutinous rice flour, semi-dry milling, lactic acid bacteria fermentation, physicochemical properties, textural quality, volatile flavor compounds

## Abstract

This study selected three lactic acid bacteria strains (*Lactiplantibacillus plantarum* CGMCC 1.12974, *Limosilactobacillus fermentum* CICC 22704, and *Lactobacillus acidophilus* CICC 22162) to pre-ferment brown and polished glutinous rice grains followed by semi-dry milling, aiming to systematically compare the differential effects of various strain treatments on the structure, functionality, and flavor of glutinous rice flour. Pre-fermentation decreased protein content (11.23–20.80% in polished flour, 0.14–2.45% in brown flour), enriched γ-aminobutyric acid (GABA, up to 6.94 mg/100 g), increased relative crystallinity and gelatinization enthalpy, and altered hydration properties. Dumplings from fermented flours showed higher soup transmittance (up to 44.93% in the brown rice group), reduced hardness (24.82–67.32%) and chewiness, and improved cohesiveness and resilience. Volatile profiles shifted towards higher acids and esters and lower alcohols, aldehydes and ketones, with strain-specific features. Redundancy analysis confirmed that starch became the dominant texture-determining factor after fermentation. Among the three strains, *Lp. plantarum* CGMCC 1.12974 exhibited the best improvement for most quality attributes. In conclusion, pre-fermentation of rice grains effectively enhances whiteness, GABA content, textural softness, and flavor diversity of semi-dry milled glutinous rice flours.

## 1. Introduction

Glutinous rice (*Oryza sativa* L. var. *glutinosa*), characterized by an exceptionally high amylopectin content (typically > 98% of total starch), exhibits unique pasting and gelling properties, making it an important raw material for traditional rice-based foods such as glutinous rice dumplings (tangyuan), rice cakes, steamed rice cakes, and sticky rice dumplings (zongzi) [1]. With the continuous growth of the convenience and frozen food market, the development of industrial products based on glutinous rice flour (GRF) has attracted increasing attention [2]. However, the processing method of GRF directly determines its physicochemical properties and the quality of the final products.

Semi-dry milling, a recently developed milling technology, combines the advantages of dry and wet milling by moderately humidifying the grains before grinding. It reduces mechanical damage through water lubrication while avoiding the high energy consumption and water pollution associated with wet milling [3,4]. Studies have shown that semi-dry milled GRF prepared at 33% moisture content confers properties close to those of wet-milled flour and comparable quality of tangyuan by reducing starch damage and preserving starch granule integrity [5]. However, existing optimization of semi-dry milling has mainly focused on parameters such as conditioning moisture, soaking conditions (time, temperature, etc.), and drying temperature [6,7,8]. There are inherent limitations in semi-dry milling for further improving the functional quality of GRF. Therefore, it is necessary to pre-treat the raw materials before milling to directionally enhance the quality of semi-dry milled GRF.

Microbial fermentation is a mild, green, and efficient bioprocessing technology. Among various microorganisms, lactic acid bacteria (LAB) represent one of the most frequently employed microbial groups in cereal fermentation. Through acid production, enzymatic action, and metabolic transformation, LAB drive major shifts in the chemical, microstructural, functional, and flavor dimensions of cereals. In the field of rice processing, fermentation has been employed to improve the pasting, retrogradation, digestibility, cooking performance, and palatability of ready-to-eat rice and rice noodle products [9,10,11]. From the perspective of compositional changes, LAB fermentation lowers the levels of protein and fat in rice flour, reorganizes the starch molecular structure, and modulates both the total starch content and the amylose/amylopectin ratio [12,13]. Furthermore, fermentation promotes the release of bound compounds, increases the availability of bioactive components, and activates glutamate decarboxylase, resulting in a substantial accumulation of γ-aminobutyric acid (GABA), a bioactive amino acid recognized for its blood pressure-lowering, anxiety-reducing, and other physiological benefits [14,15]. In terms of flavor, LAB fermentation also produces characteristic volatiles, including aldehydes, esters, alcohols, and ketones, which confer a distinctive fermented aroma to the products [11]. Together, these results confirm that LAB fermentation effectively modifies the quality of rice flour.

Most studies investigating how LAB fermentation affects rice flour have adopted the “milling-before-fermentation” sequence, i.e., rice is milled first, and the resulting flour is subsequently fermented. Although this sequence facilitates mass exchange during fermentation, the large specific surface area of the flour may lead to excessively rapid reaction rates, and the liquid fermentation-drying-remilling process in industrial production increases energy consumption [10]. In contrast, the alternative “fermentation-of-grains-before-semi-dry-milling” route may induce changes in rice grain structure and composition prior to milling, thus potentially combining the biochemical effects of fermentation with the physical actions of milling, yet research on its effects on GRF quality remains limited, particularly regarding the differential responses between brown rice (with bran retained) and polished rice (with bran removed). To address these knowledge gaps, this study aimed to: (1) systematically evaluate the effects of grain pre-fermentation with three LAB strains on the physicochemical properties, processing quality, and flavor of semi-dry milled GRF; (2) compare the differential effects between brown and polished glutinous rice; and (3) elucidate, via redundancy analysis and other approaches, the changes in key compositional factors governing the textural properties of GRF after fermentation.

## 2. Materials and Methods

### 2.1. Materials

In line with our previous work, both brown and polished glutinous rice of the cultivar ‘Longjing 57’ were used as raw materials. Brown rice was obtained by dehulling the paddy using a rice huller (FC2K, Otake Seisakusho, Sakado, Japan), and polished rice was further processed from the brown rice using a rice polisher (VP-32, Yamamoto Seisakusho, Sakai, Japan). The three LAB strains used as starter cultures are listed in Table 1. All strains were activated in MRS liquid medium at 37 °C with a 2% (*v*/*v*) inoculum for three generations. The pre-fermentation method reported in [16] was adopted with modifications: equal cell concentrations (10^8^–10^9^ CFU/mL) of each strain were added (2%, *v*/*m*) to 250 mL flasks, each containing 50 g of rice and 75 mL of sterile distilled water. After thorough mixing, the mixtures were kept static at 37 °C for 24 h. To monitor the growth and metabolic status of each strain during fermentation, the initial and final LAB counts, final pH, and final moisture content of the fermentation systems were determined (Table S1). LAB counts were measured according to the pour-plate method described in GB 4789.35-2016 [17]. Final pH was measured at room temperature using a pH meter (S210-K, Mettler Toledo,Greifensee, Switzerland) from the supernatant of the fermentation broth. Final moisture content was determined by drying the fermented rice grains at 105 °C to constant weight and calculated by the weight loss method. After fermentation, the samples were rinsed twice with distilled water, drained, then pulverized for 2 min using a universal grinder (M 20 Universal mill, IKA, Staufen, Germany) at 20,000 rpm, and finally dehydrated at 40 °C to a moisture content of <10% (*w*/*w*).

### 2.2. Chemical Composition

The contents of total starch, damaged starch, protein, reducing sugar, total acidity, total phenols, and total flavonoids were determined according to our previous study [16]. GABA was quantified by HPLC-UV after DABC-SCl derivatization. Samples were extracted twice with 80% ethanol under ultrasonication, and the combined supernatants were adjusted to 25 mL. An aliquot (1.0 mL) was derivatized with 0.20 mL sodium bicarbonate (40 g/L) and 0.40 mL DABC-SCl (2 mg/L) at 70 °C for 20 min. Separation was carried out on an Ultimate 3000 HPLC system (Thermo Scientific, Waltham, MA, USA) equipped with a C18 column (Agilent, Santa Clara, CA, USA, 4.6 × 250 mm, 5 µm) maintained at 30 °C, using acetonitrile-sodium acetate (6.8 g/L) (35:65, *v*/*v*) at 1.0 mL/min, with UV detection at 436 nm. Quantification was performed using an external standard curve.

### 2.3. Physicochemical and Structural Attributes of GRF

#### 2.3.1. Color Properties

Using a spectrophotometer (CM5, Konica Minolta, Inc., Tokyo, Japan), the L, a, b color parameters of GRF were determined, and the Hunter whiteness index was calculated according to our previous study [16].

#### 2.3.2. Water Absorption Index, Solubility, and Swelling Power

The measurement procedure was as follows. A 0.1 g GRF sample (W_0_) was placed in a pre-weighed centrifuge tube (W_1_) containing 20 mL deionized water. The tube was agitated in a water bath at 25 °C for 30 min, and separately at 100 °C for 30 min. After centrifugation (2000× *g*, 30 min), the supernatant was collected in a pre-weighed aluminum dish (W_2_) and dried at 105 °C to constant mass (W_3_). The weight of the tube with the wet sediment was recorded (W_4_).

Water absorption index (WAI) = (W_4_ − W_1_)/W_0_

Water solubility index (WSI) = (W_3_ − W_2_)/W_0_

Swelling power (SP) = (W_4_ − W_1_)/[W_0_ × (1 − WSI)]

#### 2.3.3. Scanning Electron Microscopy (SEM)

A scanning electron microscope (Sigma 300, ZEISS, Oberkochen, Germany) was used to characterize the morphology of the GRF samples. The specimens were attached to aluminum stubs with conductive tape and gold-coated for surface conductivity. High-vacuum mode was applied during image acquisition, and images were captured at an accelerating voltage of 3 kV and a magnification of 1000×.

#### 2.3.4. Powder X-Ray Diffraction (XRD)

XRD patterns were collected on an X-ray diffractometer (Ultima IV; Rigaku Corporation, Osaka, Japan) to analyze the crystalline characteristics of each rice flour sample. All samples exhibited comparable moisture content and particle size prior to analysis. The 2θ angle was scanned from 5° to 40° at 2°/min. Relative crystallinity (%) was calculated with Jade 6 (Materials Data, Inc., Livermore, CA, USA) by subtracting the amorphous halo area from the total peak area.

#### 2.3.5. Differential Scanning Calorimetry (DSC)

A DSC Q2000 (TA Instruments, New Castle, DE, USA) was used for thermal analysis of GRF. Briefly, 6 mg of sample was mixed with deionized water (1:2, *w*/*v*) in a stainless steel pan. The sealed pans were then stored at 4 °C for 24 h. The heating program was set to ramp from 20 °C to 100 °C at 5 °C/min, with an empty pan serving as reference.

#### 2.3.6. Rapid Visco Analysis (RVA)

Pasting properties of GRF were determined using a Brookfield DV2T viscometer (Brookfield Engineering Laboratories, Inc., Middleboro, MA, USA) following a previously reported procedure with minor adjustments. A flour suspension (8%, *w*/*w*) was prepared by dispersing 3 g of GRF in deionized water. The temperature program used for pasting is shown in Table S2.

### 2.4. Preparation of Glutinous Dumpling

To prepare the dough, water was added at a level of 90% relative to the flour mass. The mixture was enclosed in plastic wrap. Thereafter, a 3.0 g aliquot was manually shaped into a ball, enveloped tightly in polyethylene film, and subsequently frozen at −20 °C for a period of seven days.

### 2.5. Evaluation of Glutinous Dumpling Quality

Soup transparency was evaluated at 620 nm. After cooking five dumplings in 500 mL of boiling water for 6 min, the liquid was cooled and adjusted back to 500 mL. Its absorbance was then measured with a UV-vis spectrophotometer (CARY 100, Varian, Inc., Palo Alto, CA, USA) using distilled water as a reference.

To determine cooking loss, a 15 mL aliquot of the soup was transferred to a pre-weighed aluminum dish and dried at 105 °C to constant weight to determine the dry matter content of the soup. Cooking loss was calculated as follows:

Cooking loss (%) = [(dry matter mass of soup × 500/15)/total mass of raw dough] × 100%

The textural properties of cooked dumplings (room temperature) were determined with a TA-XT Plus analyzer (Stable Micro Systems, Godalming, UK) equipped with a P/36R probe. The settings were as follows: 2.0 mm/s (pre-test), 1.0 mm/s (test), 1.0 mm/s (post-test), 50% deformation, 5 s waiting time between two compressions, and 5 g trigger force.

### 2.6. Volatile Components Analysis

Volatile compounds were extracted by headspace solid-phase microextraction (HS-SPME) and analyzed by gas chromatography-mass spectrometry (GC-MS). A 2 g sample was placed in a 20 mL headspace vial containing 3 mL of saturated NaCl solution spiked with an internal standard (2-octanol, 10 mg/L in water) at a 100:1 volume ratio. A StableFlex SPME fiber (50/30 µm DVB/CAR/PDMS, 2 cm; Supelco, Inc., Bellefonte, PA, USA) was used to trap volatiles at 60 °C for 30 min. Desorption was carried out at 250 °C for 5 min in splitless mode. The GC-MS system (Agilent 7890B-5977B, Agilent Technologies, Inc., Santa Clara, CA, USA) was equipped with a DB-Wax column (30 m × 250 µm × 0.25 µm). Helium (1.0 mL/min) was the carrier gas. The oven temperature program is detailed in Table S2. The ion source, injector, and transfer line were maintained at 230 °C, 250 °C, and 250 °C, respectively. Mass spectra were recorded at 70 eV (EI) over m/z 20–400 (full scan). Compound identification relied on the NIST library, and relative quantification was performed with the internal standard.

### 2.7. Statistical Analysis

Data were analyzed with GraphPad Prism 8 (GraphPad Software, San Diego, CA, USA) and SPSS Statistics 19.0 (IBM Corp., Chicago, IL, USA). One-way ANOVA with post hoc Waller-Duncan’s test was applied to detect significant differences between groups. In figures, different superscript letters indicate significant differences between groups (*p* < 0.05), whereas the same letters indicate no significant difference (*p* > 0.05). The vegan package (v2.5-7) was employed for redundancy analysis (RDA) to assess how much of the sample texture variance could be explained by their basic chemical composition. Additionally, permutational multivariate analysis of variance (PERMANOVA) was used to verify the significance of the observed differences. After Z-score normalization, the peak areas of the identified volatile compounds were visualized as heatmaps using an online platform (http://bioinformatics.com.cn/plot_basic_cluster_heatmap_plot_024). SIMCA (V14.1, Sartorius Stedim Data Analytics AB, Umea, Sweden) was employed to perform principal component analysis (PCA) for comparing the volatile profiles of the different samples. Variable importance in projection (VIP) scores, reflecting variable importance in projection, were obtained from partial least squares discriminant analysis modeling of the volatile compound data. Compounds with VIP > 1.0 were deemed to contribute significantly to the separation between different groups and were thus selected for further analysis. Pearson correlations between characteristic differential volatile compounds (VIP score top ten) and textural properties were analyzed. Correlation networks were then constructed using Gephi (v0.9.2) (*p* < 0.05).

## 3. Results and Discussion

### 3.1. Chemical Composition of Different GRF

**Figure 1:**
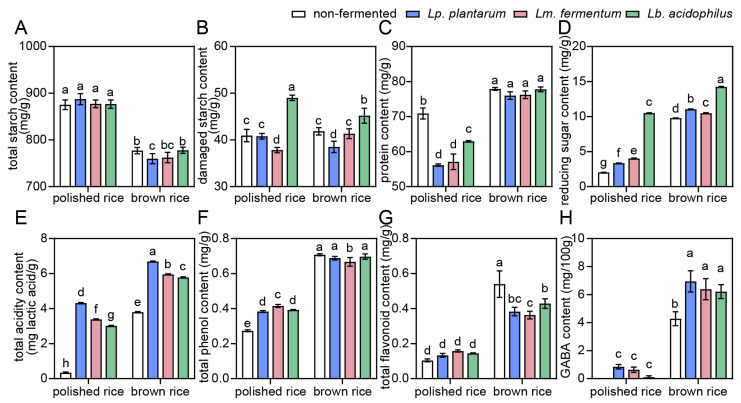
Chemical composition of non-fermented and fermented PGRF and BGRF. (**A**) Total starch; (**B**) Damaged starch; (**C**) Protein; (**D**) Reducing sugar; (**E**) Total acidity; (**F**) Total phenolics; (**G**) Total flavonoids; (**H**) GABA. All strain designations are as listed in Table 1. Different lowercase letters indicate significant differences at *p* < 0.05, same letters indicate no significant difference.

Before fermentation, brown glutinous rice flour (BGRF) and polished glutinous rice flour (PGRF) (semi-dry milled) showed distinct chemical composition profiles (Figure 1). Generally, the contents of protein (77.91 vs. 70.89 mg/g), reducing sugar (9.78 vs. 2.03 mg/g), total acidity (3.80 vs. 0.34 mg/g), total phenolics (0.71 vs. 0.27 mg/g), total flavonoids (0.54 vs. 0.10 mg/g) and γ-aminobutyric acid (GABA, 4.28 vs. not detected mg/100 g) were significantly higher in BGRF than in PGRF (*p* < 0.05), which is attributed to the nutrient-rich bran layer retained in brown rice [18]. BGRF exhibited a slightly higher damaged starch content than PGRF (41.85 vs. 40.95 mg/g) because the hard and fiber-rich bran layer of brown rice required greater mechanical force during milling, causing more severe physical damage to starch granules. In contrast, the total starch content of PGRF was slightly higher (875.55 vs. 777.18 mg/g), reflecting the removal of bran and the consequent reduction in non-starch components [19]. These baseline differences set the stage for discussing the fermentation-induced changes below.

The changes in chemical composition caused by LAB fermentation were highly dependent on the degree of milling and the bacterial strain. After fermentation, total starch (Figure 1A) showed minor numerical increases (0.13–1.36%) in PGRF compared to the non-fermented control, with no statistically significant difference (*p* > 0.05). In BGRF, total starch content showed a slight numerical decrease in all fermented samples; however, only the *Lp. plantarum* CGMCC 1.12974-fermented group exhibited a statistically significant decrease (*p* < 0.05), while the other two strains showed no significant difference from the non-fermented control (*p* > 0.05). The slight increase in fermented PGRF may be due to a relative enrichment effect: fermentation concurrently removed other components (protein, fats, minerals), thereby increasing the relative starch content [20]. In fermented BGRF, the slight decrease indicated that limited starch hydrolysis by bacterial amylases marginally exceeded the enrichment effect, as previously reported by Yang et al. on brown rice kernels fermented with LAB [10]. Damaged starch (Figure 1B) exhibited clear strain differences after fermentation. *Lp. plantarum* CGMCC 1.12974 showed a numerical reduction in PGRF (0.36%, *p* > 0.05) but significantly reduced damaged starch in BGRF (7.61%, *p* < 0.05). *Lm. fermentum* CICC 22704 significantly reduced damaged starch in PGRF (8.00%, *p* < 0.05), while its reduction in BGRF (1.24%) was not significant (*p* > 0.05). In contrast, *Lb. acidophilus* CICC 22162 led to a significant increase in both PGRF and BGRF by 19.71% and 8.01%, respectively (*p* < 0.05). Protein content (Figure 1C) significantly decreased in fermented PGRF (by 11.23–20.80% relative to non-fermented PGRF) (*p* < 0.05), while it showed only minor numerical decreases in fermented BGRF (by 0.14–2.45%) with no statistically significant differences (*p* > 0.05), owing to proteolysis by LAB [21,22,23]. LAB proteolysis is mediated by cell-envelope proteinases that hydrolyze rice proteins into peptides and amino acids for bacterial assimilation [24]. Among the three strains, *Lp. plantarum* CGMCC 1.12974 caused the largest decrease, consistent with its strong proteolytic system [25]. The smaller change in fermented BGRF may be due to the intact bran layer acting as a physical barrier that restricted microbial access to endosperm proteins; in fermented PGRF, the exposed protein matrix was readily degraded by LAB proteases. Reducing sugars (Figure 1D) and total acidity (Figure 1E) significantly increased in all fermented samples (*p* < 0.05) compared to their non-fermented counterparts. The increase in reducing sugars after fermentation was attributable to the hydrolysis of starch and cellulose, while the efficient conversion of fermentable sugars into lactic acid directly caused the rise in total acidity [26,27]. Total phenolics (Figure 1F) and total flavonoids (Figure 1G) slightly increased in fermented PGRF (by 40.23–52.01% vs. non-fermented), but slightly decreased in fermented BGRF (by 1.44–5.74%). In fermented PGRF, this small increase likely resulted from the partial release of bound phenolics and flavonoids into free forms upon fermentation [28]. In fermented BGRF, despite the abundance of bound phenolics in the bran layer, the net content decreased, indicating that microbial metabolism or chemical oxidation of released phenolics, or their leaching into the discarded fermentation broth, exceeded the release. GABA (Figure 1H) was not detected in non-fermented PGRF, but after fermentation, it accumulated to 0.11–0.85 mg/100 g in fermented PGRF; in BGRF, fermentation increased GABA content by 45.16–62.25% compared to non-fermented BGRF (*p* < 0.05). Among the strains, *Lp. plantarum* CGMCC 1.12974 gave the highest GABA content after fermentation, reaching 0.85 mg/100 g in fermented PGRF and 6.94 mg/100 g in fermented BGRF. The marked increase in GABA after fermentation reflects the robust glutamate decarboxylase activity of the three LAB strains, which catalyzes the conversion of L-glutamate to GABA—a well-established pathway in cereal fermentation [29]. GAD is a PLP-dependent enzyme that exhibits higher activity under acidic conditions [30]. The higher total acidity in *Lp. plantarum* CGMCC 1.12974-fermented samples (Figure 1E) may have provided a favorable condition for GAD activity, partly explaining its highest GABA yield. Notably, the accumulation of GABA in fermented BGRF was generally higher than that in fermented PGRF, most likely due to the higher initial glutamate content in the bran layer, providing a richer substrate pool for GABA synthesis [31]. In terms of percentage increase, the GABA enhancement in BGRF (45.16–62.25%) in this study was close to the 49.0% increase reported by Kwon et al. in LAB solid-state fermentation of brown rice, falling within the range of conventional fermentation reported in the literature [32]. In contrast, Tram et al. achieved a 141-fold increase (from 0.13 to 24.01 mg/100 g) in Mang Buk brown rice under optimized conditions—demonstrating that fermentation conditions significantly influence GABA enrichment efficiency and suggesting that further optimization could enhance the GABA yield in the present system [33].

### 3.2. Physicochemical Properties of Different GRF

#### 3.2.1. Color Properties and Whiteness

The color parameters and Hunter whiteness values of BGRF and PGRF are shown in Figure S2. Before fermentation, the L* and Hunter whiteness values of PGRF (95.32 and 92.72) were significantly higher than those of BGRF (88.62 and 83.89) (*p* < 0.05) (Figure S1A,D). Notably, the a* value of PGRF was negative (greenish tone), whereas that of BGRF was positive (reddish tone) (Figure S1B). Tuncel et al. also reported that with increasing degree of milling and consequent bran removal, lightness and whiteness increase, yellowness decreases, and all but polished rice exhibit a slight red tone [34]. The rice pigments, mainly concentrated in the bran and aleurone layers, are progressively stripped away during the conversion of brown rice into polished rice [35].

LAB fermentation induced significant changes in color parameters (*p* < 0.05). In BGRF, after fermentation, L* and Hunter whiteness significantly increased by 1.38–2.15% and 1.78–2.90%, respectively, indicating a clear brightening effect. Meanwhile, a* significantly decreased by 7.67–11.67%, and b* decreased by 7.77–13.52% (*p* < 0.05) (Figure S1A–D). In PGRF, fermentation also caused significant changes: L* and whiteness increased by 0.39–1.07% and 0.63–1.63%, respectively; b* significantly decreased by 7.91–19.89%; and a* increased by 42.24–46.55% but remained negative (*p* < 0.05). These changes are attributed to the fermentation-induced modifications of starch and protein components, thereby modifying their light-scattering behavior and ultimately causing color shifts [36].

Notably, the magnitudes of increase in L* and whiteness were greater in BGRF than in PGRF (Figure S1D). The reason lies in the higher initial pigment load and lower baseline whiteness of brown rice, providing greater room for fermentation-induced brightening. Among the three strains, *Lp. plantarum* CGMCC 1.12974 induced the greatest increase in whiteness in BGRF, followed by *Lm. fermentum* CICC 22704 and *Lb. acidophilus* CICC 22162 (Figure S1D). As discussed in Section 3.1, this strain-dependent ranking aligns with the extent of protein reduction. Previous studies have indicated that a lower protein content can significantly increase L* value [37]. Therefore, the stronger proteolytic capacity of *Lp. plantarum* strain likely contributed to its superior whitening effect. Overall, fermentation effectively brightened the color of both flours.

#### 3.2.2. Water Hydration Properties

Before fermentation, the WAI (3.46 g/g and 15.00 g/g at 25 °C and 100 °C, respectively) and SP (3.39 g/g and 10.49 g/g) of PGRF were slightly higher than those of BGRF (WAI: 3.34 g/g and 13.56 g/g; SP: 3.04 g/g and 9.73 g/g), whereas the WSI of BGRF (8.91% and 31.19%) was slightly higher than that of PGRF (1.85% and 30.31%) (Figure S2). The lower WAI and SP of BGRF, consistent with the report of Qiu et al. [38], can be ascribed to its higher protein and lipid contents. Proteins and lipids in BGRF suppress starch swelling and granule disruption, thus reducing water-holding and swelling capacities [39]. Soluble components (free sugars, soluble dietary fiber, minerals) present in the bran layer are largely responsible for the higher WSI of BGRF, as these are mostly removed during the polishing of rice. Hot water yielded significantly higher values for all hydration properties than cold water did (*p* < 0.05). This phenomenon can be explained by the disruption of crystalline structure during heating, which affects hydrogen bonds associated with water, amylose, and amylopectin branches, thereby promoting WAI and SP of starch. Thermal activation enhances molecular mobility and facilitates the dispersion of soluble components, thus amplifying WSI values [40].

Fermentation caused a decrease in WAI through enzymatic hydrolysis of starch and proteins, thereby lowering water-holding capacity [41]. It also reduced SP, likely by debranching amylopectin and increasing short-chain content, which restricts granule swelling—a finding consistent with the notion that disrupted chemical bonds impair starch swelling [42,43]. The increased proportion of short-chain amylopectin forms more stable crystalline structures that are less prone to swelling upon heating [42]. In contrast, fermentation raised WSI, as microbial enzymes degraded insoluble components (proteins and cell wall polysaccharides) into soluble substances, while the protein network collapse allowed starch to leach into the aqueous phase [44,45].

#### 3.2.3. Microstructure

**Figure 2:**
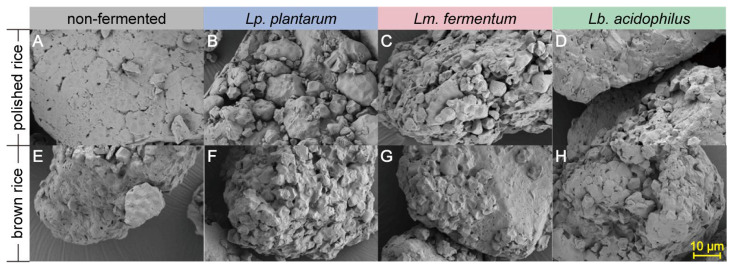
SEM images of non-fermented and fermented GRF. (**A**) Non-fermented PGRF; (**B**) *Lp. plantarum* CGMCC 1.12974-fermented PGRF; (**C**) *Lm. fermentum* CICC 22704-fermented PGRF; (**D**) *Lb. acidophilus* CICC 22162-fermented PGRF; (**E**) Non-fermented BGRF; (F) *Lp. plantarum* CGMCC 1.12974-fermented BGRF; (**G**) *Lm. fermentum* CICC 22704-fermented BGRF; (**H**) *Lb. acidophilus* CICC 22162-fermented BGRF.

As shown in Figure 2, all rice flours exhibited a compact microstructure, in which polyhedral starch granules (typically 3–8 μm in diameter) were embedded in a continuous protein matrix [46]. After LAB fermentation, both BGRF and PGRF exhibited a greater quantity of small granules, along with rough surfaces and pores on some granules. The surface modifications observed after LAB fermentation stem from microbial protease-mediated hydrolysis of granule-associated proteins. These structural defects, in turn, enhance enzyme accessibility and accelerate the breakdown of internal granule constituents [47,48]. Moreover, fermentation induced fragmentation of BGRF into finer particles, leading to a reduction in particle size [10]. These structural modifications also explain the increased whiteness of fermented flours. First, surface pores and fine particles scatter light more effectively. Second, removal of the protein matrix exposes the underlying starch granules. Together, these changes enhance total light reflectance, leading to higher Hunter whiteness values.

#### 3.2.4. Crystalline Structure

**Figure 3:**
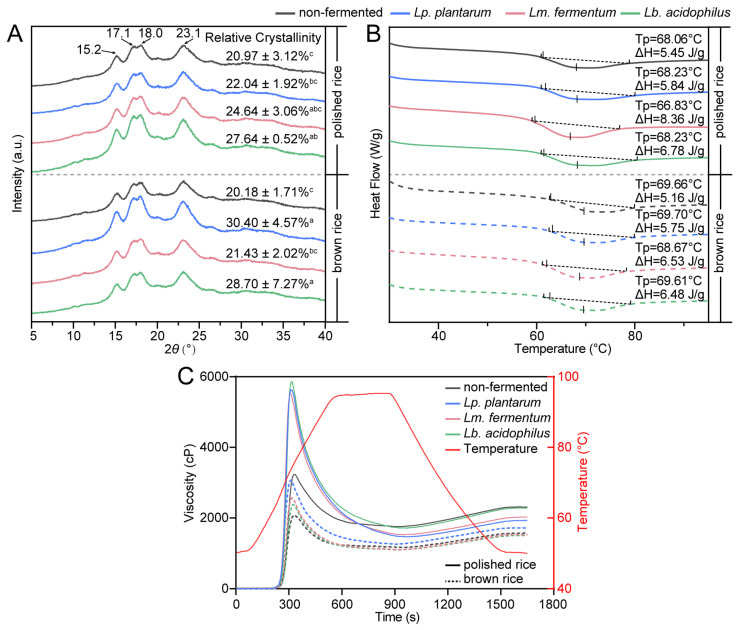
Crystalline structure, thermal properties and pasting behavior of non-fermented and fermented PGRF and BGRF. (**A**) XRD patterns; (**B**) DSC thermograms; (**C**) RVA pasting curves. All strain designations are as listed in Table 1. Different lowercase letters indicate significant differences at *p* < 0.05, same letters indicate no significant difference.

The XRD patterns of all samples displayed the typical A-type starch features, with two single peaks at 15.2° and 23.1° (2θ) and a doublet at 17.1° and 18.0° (2θ) [49]. The relative crystallinity of non-fermented PGRF and BGRF did not differ significantly (*p* > 0.05) (Figure 3A).

Previous studies have also shown that fermentation does not alter the crystalline pattern of rice starch [9,50]. However, fermentation treatment increased the relative crystallinity of both PGRF and BGRF. The relative crystallinity values of non-fermented PGRF and BGRF were 20.97% and 20.18%, respectively, which increased to 22.04–27.64% and 21.43–30.40% after fermentation. The rise in relative crystallinity probably results from the selective breakdown of amorphous starch fractions by LAB-derived enzymes or organic acids, which in turn raises the crystalline fraction [51].

#### 3.2.5. Thermal Properties

The enthalpy of gelatinization (ΔH) is the endothermic energy required for disintegrating the crystalline structures within starch granules during heating, and it is strongly dependent on the degree of starch crystallinity (Figure 3B). The ΔH value of non-fermented PGRF (5.45 J/g) was slightly higher than that of non-fermented BGRF (5.16 J/g). This may be attributed to protein–starch gel interactions or amylose–lipid complexation [52]. Meanwhile, PGRF exhibited a slightly lower T_p_ (68.06 °C) than BGRF (69.66 °C), which is consistent with previous studies [19,53].

LAB fermentation of both PGRF and BGRF simultaneously lowered the gelatinization temperature while raising the ΔH value. The ΔH increase in fermented samples (7.16–53.39% for PGRF; 11.43–26.55% for BGRF) indicates hydrolysis of amorphous starch regions and molecular rearrangement during fermentation, leading to more ordered structures—consistent with the higher crystallinity observed (Section 3.2.4). Mao et al. reported similar results in fermented corn flour, where increased short-chain amylopectin lowered gelatinization temperature but raised ΔH through enhanced double-helix packing [54]. These modifications reinforce crystallinity and ordered helix arrangements, thus requiring more energy to disrupt crystalline zones.

#### 3.2.6. Pasting Properties

Prior reports [38,55] have shown that rice bran generally lowers pasting viscosities, and our results for non-fermented PGRF (higher PV, TV, FV, SB, and BD than BGRF) are in line with this pattern (Figure 3C). Such differences arise because proteins and lipids in rice bran physically interfere with starch swelling and water uptake, thereby limiting gelatinization and suppressing starch chain rearrangement and retrogradation upon cooling.

After fermentation, PV and BD increased in both flours, whereas the changes in TV, FV, and SB varied depending on the sample and the bacterial strain. The increase in PV may be due to the ability of LAB metabolites to disrupt the protein-starch matrix and create structural defects that favor water permeation, thereby promoting more rapid and complete starch granule swelling, ultimately leading to higher PV values [56]. This finding also corroborates the thermal properties. A higher BD value generally indicates better palatability, but it also reflects the degree of starch granule fragmentation under prolonged heating and mechanical shear, implying lower hot-paste viscosity stability and a greater tendency of swollen granules to rupture [57]. The increase in BD is likely associated with fermentation-induced weakening of the granule surface structure, which, while promoting initial water absorption and swelling, also makes the starch granules more susceptible to disintegration at high temperatures, resulting in a faster and greater viscosity drop during the holding stage.

### 3.3. Cooking Quality of Glutinous Dumplings Prepared from Different GRF

#### 3.3.1. Soup Transmittance and Cooking Loss

**Figure 4:**
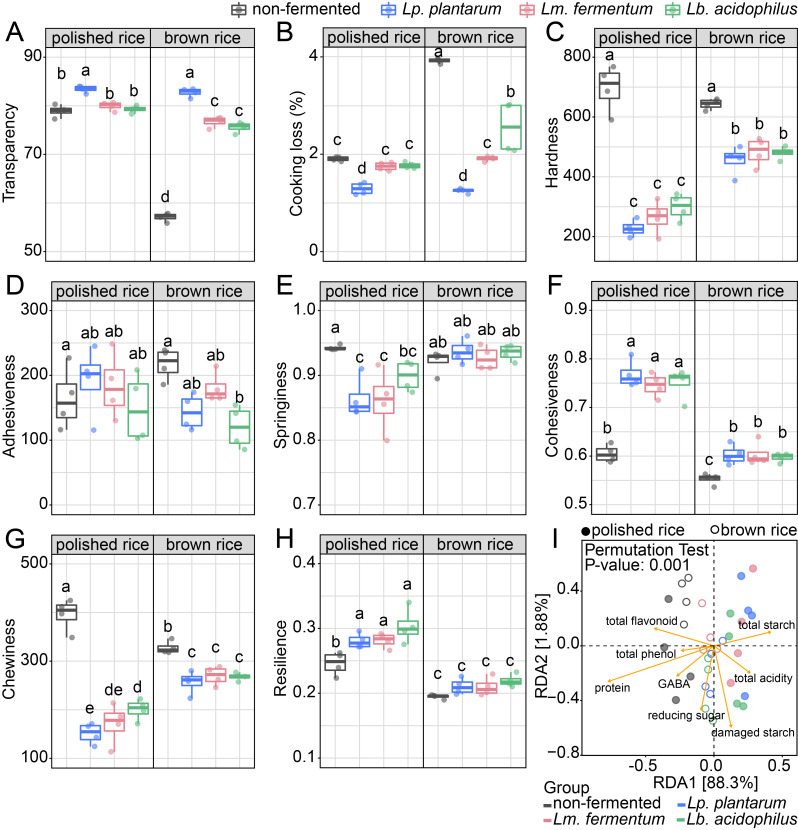
Cooking quality and textural properties of glutinous rice dumplings from non-fermented and fermented PGRF and BGRF. (**A**) Soup transmittance; (**B**) Cooking loss; (**C**–**H**) TPA parameters (hardness, adhesiveness, springiness, cohesiveness, chewiness, resilience); (**I**) RDA biplot (chemical components vs. texture). All strain designations are as listed in Table 1. Different lowercase letters indicate significant differences at *p* < 0.05, same letters indicate no significant difference.

The light transmittance of the soup after cooking glutinous rice dumplings is an important indicator of cooking quality. Higher transmittance indicates clearer soup with less solid leaching, which is preferred by consumers. As shown in Figure 4A, among non-fermented samples, the transmittance of dumplings made from PGRF was higher than that of those made from BGRF. After fermentation, the transmittance of both types of dumplings significantly increased (*p* < 0.05), with the *Lp. plantarum* CGMCC 1.12974-fermented group showing the highest values (5.73% for PGRF and 44.93% for BGRF), followed by *Lm. fermentum* CICC 22704 (1.29% and 34.35%) and *Lb. acidophilus* CICC 22162 (0.46% and 32.37%). In addition to soup transmittance, cooking loss of the dumplings was also determined (Figure 4B). After fermentation, cooking loss significantly decreased (*p* < 0.05), consistent with the increased soup transmittance. The *Lp. plantarum* CGMCC 1.12974-fermented group exhibited the lowest cooking loss (1.29% for PGRF and 1.25% for BGRF). The improvements in soup clarity and the reduction in cooking loss after fermentation are both attributable to LAB-mediated proteolysis of the protein matrix surrounding starch granules, which reduced the release of soluble solids and insoluble protein debris into the cooking water. Similarly, Geng et al. found that protein-enriched brown rice noodles showed greater cooking loss and turbidity, indicating inferior cooking performance [58].

#### 3.3.2. Textural Characteristics

The textural properties of cooked glutinous rice dumplings are a key determinant of consumer acceptance. The texture profile analysis results (Figure 4C–H) showed that before fermentation, the cohesiveness, chewiness, and resilience of the PGRF dough (0.60, 395.77, 0.25) were significantly higher than those of the BGRF dough (0.55, 327.53, 0.19) (*p* < 0.05). A comparable trend was noted in rice noodles containing rice bran, where the bran was found to suppress starch cross-linking and interfere with starch-starch molecular associations [59].

After fermentation, the hardness and chewiness of dumplings from both flours significantly decreased (*p* < 0.05). The reduction in hardness ranged from 57.04% to 67.32% for PGRF and from 24.82% to 29.17% for BGRF; the reduction in chewiness ranged from 49.35% to 61.80% for PGRF and from 17.64% to 21.65% for BGRF. Among the strains, *Lp. plantarum* CGMCC 1.12974 induced the largest decreases, followed by *Lm. fermentum* CICC 22704 and *Lb. acidophilus* CICC 22162. Fermentation partially degrades the starch-protein network, which in turn lowers hardness and chewiness, leading to a porous structure that makes the cooked gel softer and more deformable [60]. In addition, the increased WAI of fermented flours (Section 3.2.2) raised the water content of the dumplings, further contributing to texture softening. In contrast to hardness, the cohesiveness of dumplings generally increased after fermentation (Figure 4C,F), with *Lp. plantarum* CGMCC 1.12974 increasing cohesiveness by 27.01% in PGRF and 8.98% in BGRF. The increased cohesiveness after fermentation reflects a more stable gel network [61], driven by both protein removal (strengthening starch gelation) and starch molecular rearrangement (enhancing gel integrity). Resilience displayed a similar pattern (Figure 4H), ranging from 0.28 (*Lp. plantarum* CGMCC 1.12974-fermented PGRF dumplings) to 0.19 (non-fermented BGRF dumplings).

RDA was performed to explore the associations between the proximate composition of the flours and the textural properties of the dumplings. As shown in Figure 4I, the first two axes of the RDA explained 88.30% and 1.88% of the total variance, respectively, cumulatively accounting for 90.18%. PERMANOVA confirmed that the RDA model was overall significant (*p* = 0.001). The ordination plot clearly separated non-fermented samples from fermented samples along the first axis, indicating a profound impact of fermentation on textural properties. Based on the projection length and direction of the explanatory variables, protein content showed the highest correlation with the textural properties of non-fermented samples, whereas total starch content showed the highest correlation with those of fermented samples, suggesting that after fermentation, starch becomes the dominant contributor to texture.

### 3.4. Volatile Components of Different GRF

#### 3.4.1. Volatile Compound Composition

**Figure 5:**
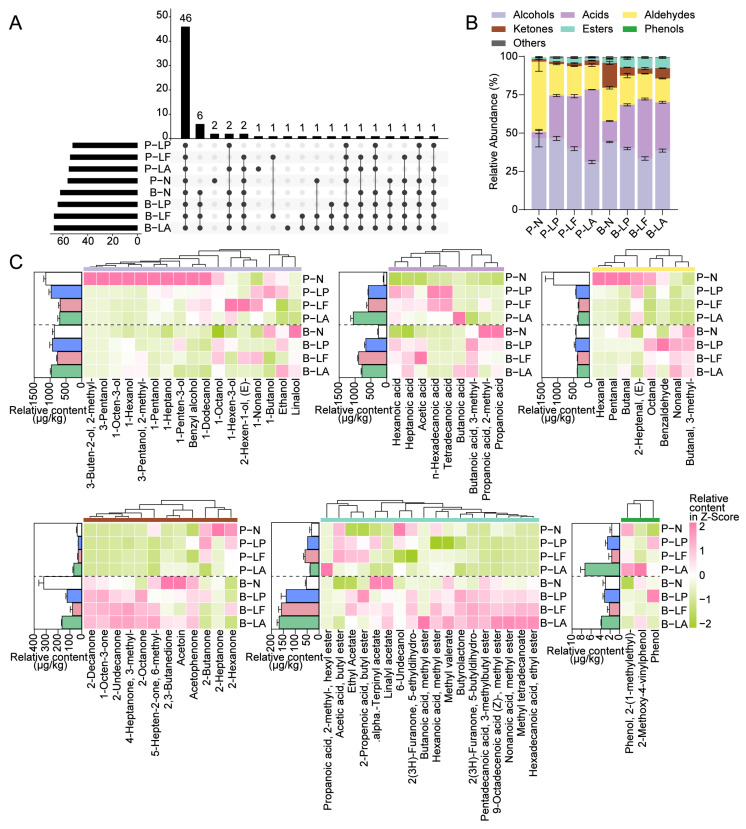
Volatile compound profiles of non-fermented and fermented PGRF and BGRF. (**A**) UpSet plot showing the distribution of unique and shared volatile compounds among samples; (**B**) Relative proportions of different chemical classes (alcohols, acids, esters, aldehydes, ketones, phenols, others); (**C**) Heatmap of representative volatile compounds. All strain designations are as listed in Table 1.

Seventy-one volatile compounds were characterized by HS-SPME/GC-MS, comprising 18 esters, 17 alcohols, 12 ketones, 9 acids, 8 aldehydes, and 3 phenols. Among them, 46 compounds were uniformly detected in all samples, and six compounds were unique to BGRF. As previously documented [62], the number and levels of volatile compounds decline with increasing milling degree. This pattern is reflected in our data, where brown rice displayed a wider range of volatiles (Figure 5A).

Alcohols had the highest proportion among all volatile classes and generally impart aromatic, plant-like, and earthy odors to rice (Figure 5B). Their proportions in non-fermented PGRF and BGRF were 46.69% and 44.19%, respectively, and decreased after fermentation to 31.12–46.47% and 33.40–39.90%, respectively. In this study, ethanol was the major alcohol, mainly derived from alcoholic fermentation via the conversion of pyruvate through the acetyl-CoA pathway or the pentose phosphate pathway of glucose (Figure 5C) [63]. Acids ranked second in proportion. Before fermentation, they accounted for only 3.94% and 13.64% in PGRF and BGRF, respectively, and increased after fermentation to 28.03–47.24% and 28.54–31.51% (Figure 5B). Fermented foods owe much of their sensory character to volatile acids, which are generated primarily through the metabolic pathways of amino acids, lactose, and lipids [64]. Moreover, the acids produced by different LAB strains varied greatly. In both PGRF and BGRF, *Lp. plantarum* CGMCC 1.12974 accumulated more hexanoic acid, *Lm. fermentum* CICC 22704 showed higher acetic acid content, and *Lb. acidophilus* CICC 22162 accumulated higher levels of butanoic acid (Figure 5C). Acids mostly impart sour and cheesy notes to foods, and excessive acid content may adversely affect sensory acceptance. Aldehydes are the main source of rice aroma, primarily generated through lipid oxidation and degradation [65]. They usually impart pleasant fresh green and light fruity notes to rice, but can also cause off-odors when present at high concentrations. The proportion of aldehydes in PGRF (45.98%) was higher than that in BGRF (21.75%), which is consistent with the finding by Lai et al. that the proportion of aldehydes increases with higher degree of milling [66]. After fermentation, the aldehyde proportion decreased to 15.96–20.55% in PGRF and to 15.57–19.07% in BGRF (Figure 5B). Fermentation likely drives the conversion of aldehydes, either by reduction to alcohols or by oxidation to organic acids [67]. Ketones, which typically contribute creamy and fruity notes, originate largely from unsaturated fatty acids via oxidation or thermal breakdown. Their proportion in BGRF (16.25%) was higher than that in PGRF (1.61%), and also decreased after fermentation. After fermentation, the proportion of esters increased substantially in both flours, from 1.29% to 1.71–3.44% in PGRF, and from 2.75% to 5.97–6.91% in BGRF. The increase may be ascribed to microbial lipid degradation yielding fatty acids, which are then esterified into volatile compounds [11]. Among them, ethyl acetate, which has fruity and sweet notes, increased after fermentation. Microbial esterification of alcohols and acids generates esters that generally confer fruity notes and suppress unpleasant odors [68]. This may also explain why the proportion of most alcohols decreased after fermentation. For example, linalool (floral, woody) decreased after fermentation, while the content of its corresponding ester, linalyl acetate, increased (Figure 5C).

**Figure 6:**
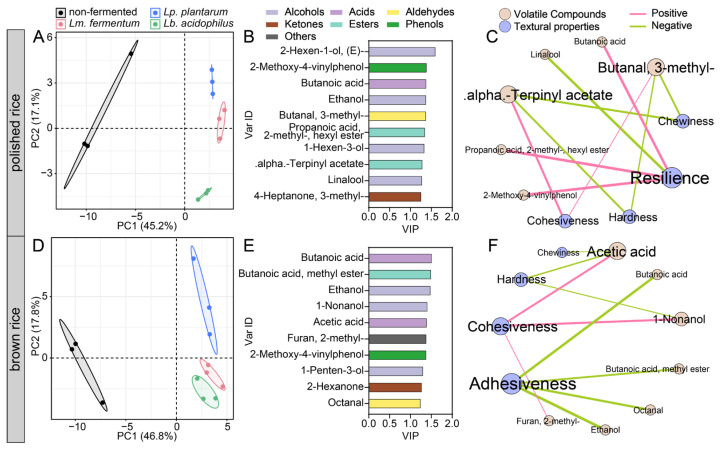
Multivariate analysis of volatiles and correlations with texture in non-fermented and fermented PGRF and BGRF. (**A**,**D**) PCA score plots (PGRF and BGRF); (**B**,**E**) VIP scores (top 10 compounds); (**C**,**F**) Correlation networks (volatiles vs. texture). Pink: positive correlation (*p* < 0.05), green: negative; line thickness indicates strength. All strain designations are as listed in Table 1.

To further clarify flavor variations among samples, PCA was applied to the PGRF and BGRF groups independently. PC1 (45.2% for PGRF and 46.8% for BGRF; Figure 6A,D) clearly distinguished non-fermented from fermented samples in both rice types, pointing to fermentation as the overriding factor shaping flavor. Based on a PLS-DA model, the top ten volatile compounds ranked by VIP scores were selected as the key discriminators among groups. In the PGRF group, these compounds encompassed various classes, including alcohols, acids, esters, aldehydes, and ketones (Figure 6B). In contrast, the BGRF group exhibited a more prominent contribution from short-chain acids (butanoic acid and acetic acid) and furans, reflecting its richer substrate pool for lipid metabolism compared to PGRF. Among them, linalool, an important quality indicator during rice aging with floral and citrus notes, decreased in content after fermentation in all cases [69]. 2-Methoxy-4-vinylphenol (clove-like, smoky) and butanoic acid (sour, rancid) were both higher in the *Lb. acidophilus* CICC 22162-fermented samples. 1-Nonanol, (E)-2-hexen-1-ol, acetic acid, and 4-heptanone, 3-methyl- were all higher in the *Lm. fermentum* CICC 22704-fermented samples.

#### 3.4.2. Correlations Between Key Volatile Compounds and Textural Properties

To explore the potential contribution of flavor compounds to the textural properties of glutinous rice dumplings, Pearson correlation analysis was performed between the characteristic differential volatile compounds described above and the TPA parameters. Resilience in the PGRF group exhibited a significant negative relationship with linalool (*p* < 0.05), while displaying positive relationships with 2-methoxy-4-vinylphenol, butanoic acid, and 2-methylpropanoic acid hexyl ester (Figure 6C). Cohesiveness was positively correlated with α-terpinyl acetate and 3-methylbutanal, while both of these compounds were negatively correlated with hardness and chewiness. This indicates that esters and aldehydes imparting floral and fruity notes help improve the springiness and internal cohesion of the dumplings while reducing excessive hardness and toughness. Notably, the contents of butanoic acid, 2-methylpropanoic acid hexyl ester, and 2-methoxy-4-vinylphenol were significantly higher in the *Lb. acidophilus* CICC 22162-fermented samples than in those fermented with other strains (*p* < 0.05), further confirming that the unique metabolic activities of this strain (e.g., ferulic acid decarboxylase and short-chain fatty acid synthesis pathways) promote the coordinated accumulation of these flavor compounds. In the BGRF group, adhesiveness was negatively correlated with ethanol; 1-nonanol exhibited a negative relationship with hardness and a positive one with cohesiveness, indicating that fatty long-chain alcohols may contribute to a harder and less cohesive texture (Figure 6F). Butanoic acid and butyric acid methyl ester were inversely related to adhesiveness, while acetic acid was inversely related to hardness and chewiness but positively related to cohesiveness. These results indicate that short-chain fatty acids produced by fermentation, especially acetic acid, help reduce product hardness and chewiness while improving internal cohesion, thereby enhancing overall textural quality.

In summary, LAB fermentation significantly reshaped the flavor characteristics of GRF by altering the contents and composition of volatile compounds, including alcohols, acids, esters, aldehydes, and ketones. More importantly, these markers showed significant correlations with the textural properties of the dumplings, suggesting that flavor formation and texture improvement may share common metabolic pathways, thereby achieving concurrent optimization of flavor and mouthfeel during fermentation.

## 4. Conclusions

This research assessed how pre-fermenting brown and polished glutinous rice with three LAB strains (*Lp. plantarum* CGMCC 1.12974, *Lm. fermentum* CICC 22704, and *Lb. acidophilus* CICC 22162) and subsequent semi-dry milling affect the chemical, functional, cooking, and volatile attributes of the resulting flours. The initial milling degree profoundly influenced the fermentation response: brown rice retained its bran layer, leading to higher levels of protein, reducing sugars, phenolics, flavonoids, and GABA, but lower brightness and hydration capacity, whereas polished rice, with the bran removed, showed higher total starch and more accessible endosperm proteins, making it more susceptible to fermentation-induced changes. Fermentation induced strain-dependent alterations in chemical components, with *Lp. plantarum* CGMCC 1.12974 exhibiting the strongest proteolytic activity and GABA accumulation, *Lm. fermentum* CICC 22704 favoring acetic acid production, and *Lb. acidophilus* CICC 22162 enriching butanoic acid and phenolic acid decarboxylation products. Structurally, fermentation preferentially degraded amorphous regions of starch, increasing relative crystallinity and gelatinization enthalpy, while disrupting the protein-starch network, which lowered water absorption and swelling power but increased water solubility. These modifications directly translated into improved cooking and textural quality of glutinous rice dumplings: soup transmittance increased markedly, hardness and chewiness decreased, and cohesiveness and resilience were enhanced, with *Lp. plantarum* CGMCC 1.12974 showing the greatest overall improvement. Redundancy analysis confirmed that the dominant factor governing texture shifted from protein to starch after fermentation. Volatile analysis revealed a comprehensive reshaping of the flavor profile, characterized by increased proportions of acids and esters and decreased proportions of alcohols, aldehydes, and ketones, with obvious strain differences. Importantly, significant correlations were identified between key volatile compounds and textural parameters, indicating that flavor formation and texture improvement share common metabolic pathways, such as proteolysis, starch rearrangement, and acid metabolism. Overall, pre-fermentation of rice grains, particularly with *Lp. plantarum* CGMCC 1.12974, represents an effective strategy to whiten the flour, enrich GABA, soften dumpling texture, and diversify flavor, supporting the development of superior fermented glutinous rice products. It should be noted that a single fermentation condition was employed in this study, with the aim of comparing the metabolic characteristics of the three strains under the same external environment. However, since the fermentation parameters were not independently optimized for each strain, these results should not be directly equated with the maximum modification capacity of each strain. Future studies may optimize fermentation conditions for different strains to further reveal their optimal modification potentials.

## Figures and Tables

**Table 1:** Lactic acid bacteria strains used in this study.

Strain	Code	Source
*Lactiplantibacillus plantarum*	CGMCC 1.12974	China General Microbiological Culture Collection Center (CGMCC)
*Lactobacillus acidophilus*	CICC 22162	China Center of Industrial Culture Collection (CICC)
*Limosilactobacillus fermentum*	CICC 22704	China Center of Industrial Culture Collection (CICC)

## Data Availability

The original contributions presented in this study are included in the article/Supplementary Material. Further inquiries can be directed to the corresponding author.

## Supplementary Materials

The following supporting information can be downloaded at https://www.mdpi.com/article/10.3390/foods15142496/s1: Table S1: Fermentation parameters of glutinous rice grains inoculated with different LAB strains. Table S2: Temperature programs for RVA pasting and GC-MS analysis. Figure S1: Color parameters and whiteness of non-fermented and fermented PGRF and BGRF. (A) *L*; (B) *a*; (C) *b**; (D) Hunter whiteness. Figure S2: Water hydration properties of non-fermented and fermented PGRF and BGRF measured at 25 °C (A–C) and 100 °C (D–F).
