# Supplementary material for: Differential Effects of Pre-Fermentation with Different Lactic Acid Bacteria Strains on the Structure, Functionality, and Flavor of Semi-Dry Milled Glutinous Rice Flour
Source: Foods. 2026 Jul 14;15(14):2496. doi: 10.3390/foods15142496 (PMC13409614; doi:10.3390/foods15142496)
Supplement: Supplementary file 1 [file foods-15-02496-s001.zip › foods-4413309-supplementary.pdf]

Differential effects of pre-fermentation with different lactic acid bacteria strains on the structure, functionality, and flavor of semi-dry milled glutinous rice flour

JingYi Zhang[1,2,3,a], Shan Shan [1,2,3,a], Shan Zhang [1,2,3], Di Yuan[1,2,3], Qi Wu[1,2,3], Bin Hong[1,2,3], ChuanYing Ren[1,2,3,*]

1 Food Processing Research Institute, Heilongjiang Academy of Agricultural Sciences, Harbin 150086, China;
18846080235@139.com (J.Z.); 18845896856@163.com (S.S.); zhangshanfood@163.com (S.Z.); yuandi199707@163.com (D.Y.); WUQI0322@163.com (Q.W.); gru.hb@163.com (B.H.)
2 Heilongjiang Province Key Laboratory of Food Processing, Harbin 150086, China
3 Heilongjiang Province Engineering Research Center of Whole Grain Nutritious Food, Harbin 150086, China
* Correspondence: chuanying1023@163.com (C.R.)
a Co-first author: JingYi Zhang, Shan Shan

Supplementary Tables

Table S1. Fermentation parameters of glutinous rice grains inoculated with different LAB strains.

Rice type	Strain	Initial LAB count (CFU/mL)	Final LAB count (CFU/ mL)	Final pH	Final moisture content (%)
Polished rice	*Lp. plantarum*	4.27×10^7	1.34×10^9	3.43	36.23
	Lm. fermentum	3.77×10^7	8.40×10^8	3.60	36.01
	Lb. acidophilus	1.20×10^7	9.00×10^7	3.76	35.48
Brown rice	*Lp. plantarum*	3.20×10^7	9.13×10^8	3.67	35.24
	Lm. fermentum	2.07×10^7	6.40×10^8	4.09	34.34
	Lb. acidophilus	1.46×10^7	6.60×10^7	4.18	33.67

Table S2. Temperature programs for RVA pasting and GC-MS analysis.

Parameter	RVA (pasting)	GC-MS (oven)
Initial hold	50 °C for 1 min	40 °C for 4 min
Heating rate	5.5 °C/min to 95 °C	5 °C/min to 245 °C
Hold at max temperature	95 °C for 5 min	245 °C for 5 min
Cooling / final	4.5 °C/min to 50 °C, then hold at 50 °C for 3 min	

Supplementary Figures

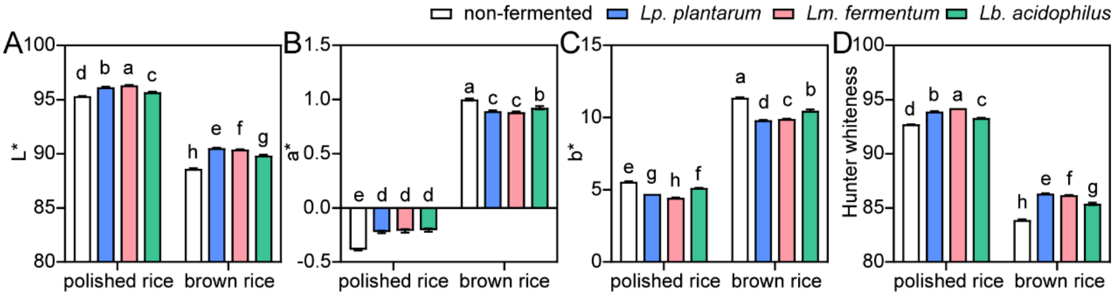

Figure S1. Color parameters and whiteness of non-fermented and fermented PGRF and BGRF. (A) *L*; (B) *a*; (C) *b**; (D) Hunter whiteness.

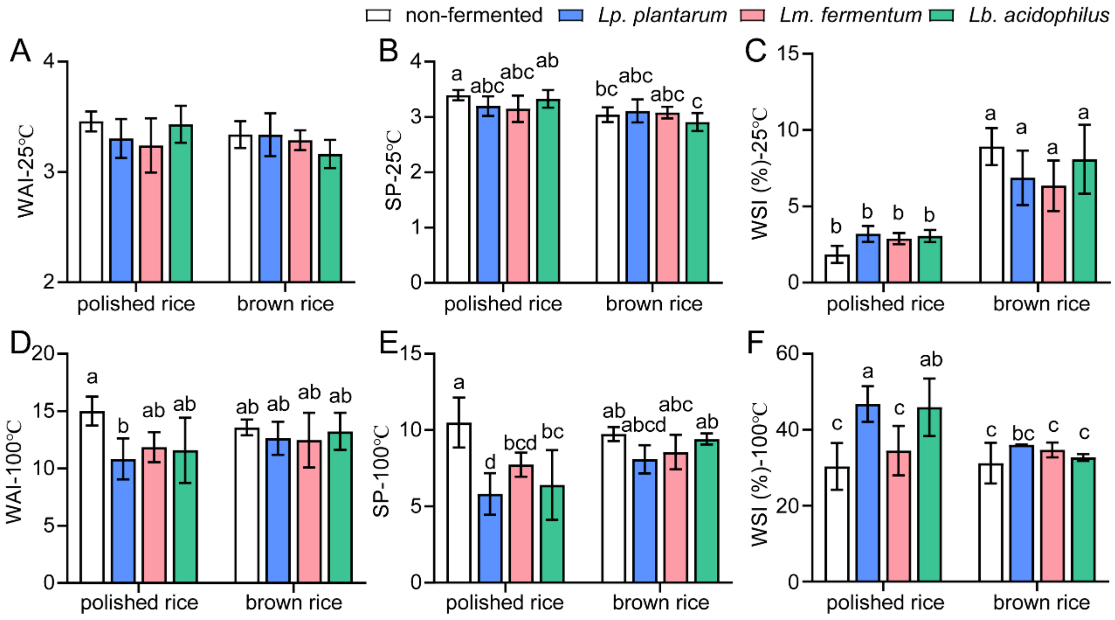

Figure S2. Water hydration properties of non-fermented and fermented PGRF and BGRF measured at 25°C (A-C) and 100°C (D-F).
